# Factors associated with the utilisation of postnatal care services among the mothers of Nepal: analysis of Nepal Demographic and Health Survey 2011

**DOI:** 10.1186/1472-6874-14-19

**Published:** 2014-01-31

**Authors:** Vishnu Khanal, Mandira Adhikari, Rajendra Karkee, Tania Gavidia

**Affiliations:** 1 Independent consultant, Maternal and Child Health, Sauraha Pharsatikar Village Development Committee, Ward 1, Rupandehi, Kathmandu, Nepal; 2 Women’s Health Project, Population Services International, Nawalparasi, Nepal; 3 School of Public Health and Community Medicine, BP Koirala Institute of Health Sciences, Dharan, Nepal; 4 Centre for International Health, Curtin University, Bentley, Western Australia

**Keywords:** Antenatal care, Determinants, Maternal mortality, Nepal, Postnatal care, Place of delivery, Wealth

## Abstract

**Background:**

Postnatal care is essential to save the life of the mother and newborn. Knowledge on the determinants of postnatal care assists the policy makers to design, justify and implement appropriate interventions. The current study aimed to analyse the factors associated with utilisation of postnatal care services by mothers in Nepal based on the data from Nepal Demographic and Health Survey (NDHS) 2011.

**Methods:**

This study utilised the data from NDHS 2011. The association between utilisation of at least one postnatal care visit (within 6 weeks of delivery) and immediate postnatal care (within 24 hours of delivery) with selected factors was examined by using Chi-square test (χ^2^), followed by multiple logistic regression.

**Result:**

Of the 4079 mothers, 43.2% reported attending postnatal care within the first six weeks of birth, while 40.9% reported attending immediate postnatal care. Mothers who were from urban areas, from rich families, who were educated, whose partners were educated, who delivered in a health facility, who had attended a four or more antenatal visits, and whose delivery was attended by a skilled attendant were more likely to report attending at least one postnatal care visit. On the other hand, mothers who reported agricultural occupation, and whose partners performed agricultural occupation were less likely to have attended at least one postnatal care visit. Similarly, mothers who were from the urban areas, from rich families, who were educated, whose partners were educated, who had attended four or more antenatal visits, who delivered in a health facility and had delivered in the presence of a skilled birth attendant were more likely to report attending immediate postnatal care. Mothers who reported agricultural occupation, and whose partners performed agricultural occupation were less likely to attend immediate postnatal care.

**Conclusion:**

The majority of postnatal mothers in Nepal did not seek postnatal care. Increasing utilisation of the recommended four or more antenatal visits, delivery at health facility and increasing awareness and access to services through community-based programs especially for the rural, poor, and less educated mothers may increase postnatal care attendance in Nepal.

## Background

The postnatal period, defined as the time immediately after the birth of the baby and up to six weeks (42 days) after birth, is critical for the newborn and the mother. Immediately after birth, bleeding and infection pose the greatest risk to the mother’s life, while preterm birth, asphyxia and severe infections pose greatest risk to newborn [1,2]. Two thirds of all neonatal deaths arise from such complications, while inappropriate feeding and cultural practices during the postnatal period may pose further risks to the life of the newborn [1,3]. All these maternal and neonatal problems could be reduced if women receive appropriate postnatal care [1]. The timing of postnatal care is also crucial to the well-being of the mother and baby. Earlier international studies have shown that some 50% of maternal deaths and 40% of neonatal deaths occur within 24 hours after birth, also known as the ‘immediate postnatal period’ [1,4]. Thus it is clear that the first 24 hours after birth is a crucial time to intervene so that any problems can be identified promptly and appropriate intervention can take place. For this reason the World Health Organisation (WHO) recommends that mothers receive postnatal care within the first 24 hours [1] followed by postnatal check on the second or third day, and then on the seventh day after delivery [1]. In Nepal, the Ministry of Health and Population, follows these recommendations [5].

Around the world, it is recognised that postnatal care is crucial in maintaining and promoting the health of the woman and the newborn baby, while providing an opportunity for health professionals to identify, monitor and manage health conditions that may develop in the mother and newborn during the postnatal period. In addition, postnatal care provides health professionals with the opportunity to promote exclusive breastfeeding, personal hygiene, appropriate feeding practices, and family planning counselling and services. Moreover, postnatal care allows for the provision of postnatal vitamin A and iron supplementation to the mother and immunisation of newborns to provide them with optimal start to life [1].

Since 1990, Nepal has made a tremendous progress in meeting the Millennium Development Goals (MDGs) related to the survival of children under five years of age and that of mothers. Between 1990 and 2010, maternal deaths have declined from 850 to 229 deaths per 100,000 live births [6]. In contrast, neonatal mortality in Nepal has reduced from 59 to 33 deaths per 1,000 live births in the same time period [5,7], remaining stagnant at 33 deaths per 1000 live births since 2006 [5]. Importantly, although postnatal care has been shown to be beneficial and effective in preventing maternal and neonatal deaths [8], attendance in Nepal remains low, with the 2011 Nepal Demographic and Health Survey (NDHS) reporting that only 46.4% of mothers attended at least one postnatal care visit [5].

A number of studies have investigated postnatal care utilisation and timing in developing countries such as Indonesia [9] and Bangladesh [10,11]. However, the literature for postnatal care in Nepal remains limited. Only two studies have reported postnatal care utilisation in Nepal [12,13]. One study conducted in 2006 [12] reported that only 34% of women utilised postnatal care service within the 42 days after birth. The same study reported that occupation, ethnicity, household economic status and education of spouse were significantly associated with the utilisation of postnatal care. The study was not without limitations. First, the study was restricted to two village development committees near the nation’s Capital, Kathmandu. Second, the sample size was small (n = 150) [12]. More recently, Neupane et al. [13] reported postnatal care utilisation in Nepal using data collected by the 2006 NDHS. The study found that only 26.5% of mothers attended at least one postnatal care visit. A literature search did not yield any other publication related to postnatal care in Nepal using data collected after 2006.

Updated knowledge using nationally representative data could provide useful information to policy makers to implement future intervention on increasing utilisation of postnatal care and improve maternal and newborn survival rates [1]. In addition, no previous studies have reported the utilisation of immediate postnatal care (within 24 hours). Given the availability of the most recent national data of NDHS 2011, and existing gaps in the literature from Nepal, this study aimed to determine the factors associated with utilisation of postnatal care within i) the first 42 days after birth, and ii) the first 24 hours after birth.

## Methods

This study analysed data from the 2011 NDHS [5], a cross sectional two- stage cluster sampling study representing the entire country. The NDHS 2011 covered the births in the five years preceding the survey. The data was collected through three sets of questionnaire: household questionnaire, men’s questionnaire and women’s questionnaire. To analyse the child health related variable, a children dataset was created by merging the relevant information from all of these three questionnaire [14]. A total of 4079 births and related information were reported within five year preceding the survey and were included in this study. Analysis was restricted to these 4079 last born children to minimise recall bias [9]. Further details of sampling, questionnaire and procedure can be found in the publicly available survey report [5]. For this study, we used publicly available dataset from the measureDHS website [14]. The NDHS 2011 is approved by the Nepal Health Research Council and ethical review board ICF Macro international. Therefore, an independent ethical approval was not required.

### Outcome variables

For this study two postnatal care utilisation outcome variables were investigated:

i) *Postnatal care attendance* was defined in this study as at least one postnatal visit provided to mother within the first 42 days (six weeks) of birth [2]. The NDHS 2011 asked a question: After you gave birth to (CHILD’S NAME), did anyone check on your health?" With options yes/no to determine if the mother had attended any postnatal care visit [5]. From the response, we used Yes = 1, and No = 0 for our analysis. There were no missing values for this response.

ii) *Immediate postnatal care* was defined in this study as postnatal care occurring within the first 24 hours of delivery. The NDHS 2011 asked "How long after delivery did the first check take place?" with response options in hours, days, weeks and do not know [5]. For analysis, responses were dichotomised into mothers who reported no postnatal care/postnatal care after 24 hours =0, and mothers who reported postnatal care within24 hours (immediate postnatal care) =1. Missing variables (n = 2 among those who responded having attended postnatal care) and no postnatal care visits were treated as late/no-postnatal care and included in the denominator to report the utilisation among the total respondents.

### Independent variables

Independent variables in this study were chosen based on existing literature on postnatal care [13,15-17]. Mother’s age was recorded as continuous variables and then recoded into categories in the NDHS dataset. Use of antenatal care (ANC) was also recorded as a continuous variable and was categorised into: (i) no ANC, (ii) 1–3 and (iii) 4 or more ANC visits. In addition, women were asked about the timing of their first ANC visit, and this was dichotomised into: (i) early ANC if the first ANC visit took place within the first trimester, and (ii) late ANC if in the second or third trimester [15]. Place of delivery was coded as (i) health facility for women who reported births in public and private health facility, the rest were classified as (ii) home deliveries [13]. Birth attendance during delivery was dichotomised into (i) skilled attendant and (ii) unskilled attendant. Doctors, nurses, health assistants, auxiliary health workers, maternal and child health workers were classified as skilled attendants; while relatives, friends and neighbours were classified as unskilled attendants [18]. Maternal final say on her own health care was included as an indicator of maternal autonomy and categorised into three groups: (i) woman alone (high autonomy), (ii) woman with partner/other (medium autonomy), and (iii) partner alone/someone else (no autonomy) [19]. Birth order was collected as a continuous variable and was recoded as (i) first (ii) second or third, and (iii) fourth or more. The economic status of each household was calculated by the NDHS using principle component analysis of more than 40 asset variables including: type of housing building materials, ownership of durable goods such as television and radio, as well as ownership of agricultural land [20]. Households were then divided into quintiles according to the wealth score: (i) poorest (ii) poor (iii) average (iv) rich and (v) richest. Considering the similarities between such quintiles as suggested by Agho et al. [21], the wealth quintiles have been re-categorised into three groups: (i) lowest 40% as poor, (ii) middle 40% as middle and (iii) upper 20% as the rich. Based on previously published NDHS related studies, ethnicity in this study was categorised according to Nepal’s caste system, which consists of more than 120 different groups, into three broad groups: (i) Advantaged, (ii) Disadvantaged Janjati and (iii) Disadvantaged Dalit [22,23]. At the top of the social order are members of the Advantaged Brahmin, Chhetri, Thakuri, Sanyasi, Gurung, and Newar groups. Members of the disadvantaged Indigenous (Janjati) population of Nepal were grouped as middle groups. The lowest position in the social order is occupied by the Disadvantaged Dalit castes [22,23]. Religion was recorded in the NDHS 2011as Hindu, Buddhist, Muslim, Christian and Kirat. As the majority (81.3%) of population belongs to Hindu religion [24], this study has dichotomised the variable into (i) Hindu and (ii) others. Education was recorded as (i) no education, (ii) primary, (iii) secondary and (iv) higher education. The same categories were used in this study. Maternal occupation was categorised into three subgroups: (i) agriculture, (ii) not working and (iii) working in paid work [22]. Paternal occupation was also recoded into three subgroups: (i) agriculture, (ii) professional/technical, and (iii) manual and others. There was no unemployed category for paternal occupation. Location of residence by development region involves the administrative division of Nepal into five vertical sections: (i) Eastern development region, (ii) Central development region, (iii) Western development region, (iv) Mid-western development region and (v) Far-western development region. Location of residence by ecological zones based on altitude divides the country into three ‘belts’ , namely (i) Mountain (ii) Hills and (iii) Terai (Plain).

### Statistical analysis

This study reports two outcome variables: (i) at least one postnatal care visit within 42 days of birth and (ii) immediate postnatal care. The association between utilisation of at least one postnatal care visit and immediate postnatal care, and the independent variables were tested by Chi-square test (χ^2^). Associations between significant variables in the Chi-square test were then further examined using crude (unadjusted) odds ratios. Significant variables in unadjusted logistic regression were then included in the multiple logistic regression models to generate adjusted odd ratios (aOR). For the multiple logistic regression analysis, a hierarchical modelling strategy is used [25]. A p-value < 0.05 was considered statistically significant. All the analyses were performed using Complex Sample Analysis procedure which was deemed necessary to adjust for sample weight, and multi stage sampling [26]. An analysis plan was prepared using strata, cluster and sample weights. This plan file was used while performing Complex Sample Analysis to achieve a more precise point and period estimation. Statistical Package for Social Science, release 20 was used for analysis.

To conceptualise the analysis, we adapted the conceptual framework which has been previously suggested by Victora et al. [27] and used in a similar study from Indonesia [28]. The framework used by the authors suggested that the utilisation of health services is affected by various factors at different levels. According to the framework, the independent variables were categorised into three main groups; starting from most distal factors: (i) community factors (place of residence, development regions, and ecological regions); (ii) sociodemographic factors (wealth status, ethnicity, religion, maternal education, maternal occupation, paternal education, and paternal occupation) and (iii) proximate factors (mother’s age, antenatal visits, timing of antenatal visits, place of delivery, attendance during delivery, maternal autonomy for health related decision, birth order and sex of child). In the multivariable analysis, we entered the most distal variables first, followed by addition of the sociodemographic variables and finally the proximate variables. Significant variables in the preceding model were retained in proceeding model, thus in model 1, we entered community factors. In model 2, we added sociodemographic factors retaining significant variables from model 1. In model 3, we retained the significant variables from model 2 and added the proximate variables. Prior to multiple regression analysis, independent variables were tested for collinearity between independent variables. A fair or weak correlation was found among the following variables: birth order and place of delivery (r = -0.33); birth order and skilled attendant (r = -0.34); maternal education and paternal education (r = 0.49); maternal education and maternal occupation (r = 0.04); paternal education and paternal occupation (r = 0.03). Skilled attendance and the place of delivery had a strong positive correlation (r = 0.89) and number of ANC visits and timing of ANC had significant correlation (r = 0.63). Similarly, mother’s age was also significantly correlated with birth order (r = 0.58). Collinearity was said to exist between place of delivery and attendance during delivery; number of ANC visits and timing of ANC visits; and mother’s age and birth order because the correlations were near to the value r = 0.85 [25]. Based on previous literature, place of delivery, number of ANC visits and mother’s age were chosen for inclusion in the final multivariate model [9,13], while attendance during delivery, timing of ANC and birth order were entered into separate model.

## Result

### Characteristics of respondents

Table 1 presents the characteristics of 4079 respondents. Almost 64% of mothers were in the age group 20–29 years, and approximately one in three women reported being first time mothers. More than a half had attended the recommended four or more ANC visits and approximately two in five women delivered their most recent birth in health facilities, while almost one in two women reported the presence of a skilled birth attendants at the time of delivery.

**Table 1:** Rate (%) of attending any postnatal care and immediate postnatal care visits by demographic and socioeconomic characteristics, Nepal 2011 (N = 4079)

**Factor**	**Total**	**Attended postnatal care**	**Attended immediate postnatal care**
	**N (%)**	**n (%)**^**#**^	**n (%)**^**#**^
**Proximate factors**			
**Mother’s age (in years)**		P < 0.001*	P < 0.001*
15-19	306 (7.5)	161 (51.6)	153 (48.9)
20-29	2608 (63.9)	1227 (46.0)	1164 (43.8)
30-34	661 (16.2)	270 (39.9)	259 (38.2)
> = 35	504 (12.4)	130 (7.7)	118 (24.3)
**Number of ANC visits**		P < 0.001*	P < 0.001*
No ANC visit	611 (15.0)	65 (11.5)	59 (10.4)
1-3	1317 (32.3)	383 (27.9)	355 (25.9)
4 or more	2151 (52.7)	1340 (63.3)	1280(60.6)
**Timing of ANC**		P < 0.001*	P < 0.001*
Late ANC (4–9 months)	2018 (49.5)	607 (30.0)	509 (35.7)
Early ANC (<=3 months)	2061 (50.5)	1181(56.5)	1126 (53.9)
**Place of delivery**		P < 0.001*	P < 0.001*
Home	2464 (60.4)	339 (14.3)	281 (11.7)
Health facility	1615 (39.6)	1449 (89.2)	1413 (87.6)
**Attendance during delivery**		P < 0.001*	P < 0.001*
Unskilled attendance	2236 (54.8)	208 (9.8)	155 (7.2)
Skilled attendance	1843 (45.2)	1580 (83.9)	1539 (82.2)
**Maternal final say on her own health care**		P < 0.001*	P < 0.001*
Women with partner/other	1522 (37.7)	737 (47.7)	707 (45.7)
Women alone	917 (22.7)	419 (44.7)	402 (43.0)
Partner alone/someone else	1594 (39.5)	616 (38.2)	571 (35.5)
**Birth order**		P < 0.001*	P < 0.001*
First	1248 (30.6)	770 (61.8)	733 (59.7)
Second or third	1847 (45.3)	802 (41.2)	758 (38.4)
Fourth or more	948 (24.1)	216 (21.6)	203 (20.3)
**Sex of child**		P = 0.209	P = 0.305
Male	2192 (53.7)	946 (42.0)	902 (39.9)
Female	1887 (46.3)	842 (44.5)	792 (42.0)
**Sociodemographic factors**			
**Wealth status**		P < 0.001*	P < 0.001*
Poor (Lower 40%)	1992 (48.8)	493 (23.8)	458 (22.2)
Middle (Middle 40%)	1416 (34.7)	756 (50.6)	723 (48.0)
Rich (Upper 20%)	671 (16.5)	539 (80.7)	213 (77.4)
**Ethnicity**		P < 0.001*	P < 0.001*
Advantaged	1955 (47.9)	971 (51.4)	921 (48.9)
Disadvantaged (Janjati)	1410 (34.6)	549 (37.4)	515 (35.2)
Disadvantaged (Dalit)	714 (17.5)	268 (35.0)	258 (33.5)
**Religion**		P = 0.008*	P = 0.014*
Hindu	3480 (85.3)	1575 (44.9)	1491 (42.5)
Others	599 (14.7)	213 (34.8)	203 (33.3)
**Maternal education**		P < 0.001*	P < 0.001*
No education	1765 (43.3)	449 (26.6)	418 (24.7)
Primary	817 (20.0)	341 (39.8)	322 (37.8)
Secondary	1225 (30.0)	760 (61.0)	728 (58.7)
Higher	272 (6.7)	238 (85.5)	226 (80.8)
**Maternal occupation**		P < 0.001*	P < 0.001*
Not working	965 (23.7)	603 (59.5)	571 (56.6)
Agriculture	2502 (61.3)	797 (31.0)	757 (29.4)
Working (paid)	612 (15.0)	388 (61.3)	366 (57.8)
**Paternal education**		P < 0.001*	P < 0.001*
No education	761 (18.7)	176 (24.2)	161 (22.3)
Primary	989 (24.2)	313 (32.3)	297 (30.2)
Secondary	1815 (44.5)	921 (50.4)	879 (48.1)
Higher	514 (12.6)	378 (74.7)	357 (71.8)
**Paternal occupation**		P < 0.001*	P < 0.001*
Agriculture	978 (24.0)	253 (26.0)	237 (24.4)
Professional/technical	1715 (42.0)	1012 (58.3)	961 (55.5)
Manual and others	1386 (34.0)	523 (37.4)	496 (35.5)
**Community factors**			
**Place of residence**		P < 0.001*	P < 0.001*
Urban	897 (22.0)	610 (71.8)	579 (68.2)
Rural	3182 (78.0)	1178 (39.9)	1115 (37.9)
**Development region**		P = 0.692	P = 0.725
Eastern	958 (23.5)	405 (46.0)	375 (42.9)
Central	854 (20.9)	381 (42.7)	364 (40.6)
Western	643 (15.8)	333 (44.4)	322 (43.1)
Mid -western	896 (22.0)	359 (38.5)	342 (36.5)
Far-western	728 (17.8)	310 (42.4)	291 (39.2)
**Ecological region**		P < 0.001*	P < 0.001*
Mountain	742 (18.2)	208 (26.7)	195 (24.9)
Hill	1656 (40.6)	663 (37.6)	628 (35.8)
Terai	1681 (41.2)	917 (49.8)	871 (47.1)

#The number of missing values may vary for each variable. The percentages presented are valid percentages. ^#^The percentage in PNC and Immediate PNC is adjusted for sample weight, multi-staging and cluster weight. Therefore, the percentage may not be equal to simple unweighted count. P = Chi-square p –value; ANC: Antenatal care; PNC: Postnatal care *reflects statistically significant association in Chi-square test.

### Utilisation of postnatal care

Less than half (43.2%; 95% CI (39.9 – 46.5%)) of the mothers had attended at least one postnatal care visit, with two in five women (40.9%; 95% CI (37.7- 44.2%)) reporting immediate postnatal care within 24 hours of delivery.

### Factors associated with postnatal care attendance

Table 1 shows the utilisation of postnatal care by selected independent variables. The study found a decrease in the proportion of mothers who attended postnatal care and immediate postnatal care services with increasing age. On the other hand, there was significant increase in postnatal care attendance with increasing ANC visits; health facility deliveries, skilled attendance, increased wealth, increased maternal and paternal educational attainments. Residing in the urban areas of Nepal was also associated with higher prevalence of postnatal care attendance and immediate postnatal care attendance. Surprisingly, only 89.2% of women reporting health facility deliveries reported any postnatal care, while only 87.6% reported immediate postnatal care.

Factors associated with the utilisation of postnatal care based on unadjusted and adjusted logistic regression are presented in Table 2. At the community level, place of residence and ecological regions were significantly associated with the attendance to postnatal care (Model 1). Mothers from urban areas (aOR 3.953; (2.992-5.222)) and from Terai (Plain) region (aOR 2.498; (1.700-3.669)) were more likely to attend postnatal care. When sociodemographic variables were added to Model 1, place of residence was the only community factor to remain significant. Of the sociodemographic factors, household wealth status, maternal education, maternal occupation, paternal education and paternal occupation were significantly associated with the postnatal care attendance after controlling for community factors, ethnicity and religion (Model 2). Mothers from middle (aOR 1.638; 95% CI (1.260-2.129)), and rich (aOR 3.182; 95% CI (2.171-4.665)) families were more likely to attend postnatal care. Similarly, mothers with education were likely to attend postnatal care than mothers with no education (aOR 4.623; 95% CI (2.880-7.421)). Fathers’ education also had positive effect on postnatal care, whereby mothers’ who had partners with higher education were more likely to attend postnatal care (aOR 1.736; 95% CI (1.099-2.742)). Mothers working in agriculture (aOR 0.623; 95% CI (0.481-0.807)) were less likely to attend postnatal care. Also mothers whose partners performed professional (aOR 1.718; 95% CI (1.354-2.179)) and manual (aOR 1.398; 95% CI (1.102-1.772)) occupations were more likely to attend postnatal care.

**Table 2:** Factors associated with attending postnatal care among Nepalese women, Demographic and Health Survey 2011

**Factor**	**Unadjusted OR (95% CI)**	**Adjusted OR (95% CI)**		
		**Model 1**	**Model 2**	**Model 3**
**Community factors**				
**Place of residence**	P < 0.001	P < 0.001	P < 0.001	P = 0.593
Rural	1.00	1.00	1.00	1.00
Urban	3.835 (2.971-4.950)	3.953 (2.992-5.222)	1.574 (1.196-2.072)	0.897 (0.603-1.336)
**Ecological region**	P < 0.001	P < 0.001	P < 0.017	P = 0.477
Mountain	1.00	1.00	1.00	1.00
Hill	1.648 (1.095-2.482)	1.422 (0.935-2.161)	1.007 (0.694-1.459)	0.898 (0.628-1.268)
Terai	2.713 (1.854-3.971)	2.498 (1.700-3.669)	1.469 (0.997-2.165)	1.374 (0.945-1.997)
**Sociodemographicfactors**				
**Wealth status**	P < 0.001		P < 0.001	P = 0.018
Poor (Lower 40%)	1.00		1.00	1.00
Middle (Middle 40%)	2.489 (1.958-3.166)		1.638 (1.260-2.129)	1.452 (1.094-1.928)
Rich (Upper 20%)	7.055 (5.469-9.056)		3.182 (2.171-4.665)	1.794 (1.103-2.917)
**Ethnicity**	P < 0.001		P = 0.735	Not in model
Advantaged	1.00		1.00	
Disadvantaged (Janjati)	0.566 (0.441-0.726)		0.921 (0.690-1.228)	
Disadvantaged (Dalit)	0.509 (0.407-0.636)		1.037 (0.808-1.332)	
**Religion**	P = 0.008		P = 0.170	Not in model
Hindu	1.525 (1.115-2.086)		1.263 (0.899-1.774)	
Others	1.00		1.00	
**Maternal education**	P < 0.001		P < 0.001	P = 0.049
No education	1.00		1.00	1.00
Primary	1.826 (1.453-2.296)		1.469 (1.180-1.828)	1.092 (0.809-1.476)
Secondary	4.320 (3.402-5.482)		2.279 (1.754-2.961)	1.153 (0.823-0.823)
Higher	16.247 (10.309-25.605)		4.623 (2.880-7.421)	2.257 (1.193-4.267)
**Maternal occupation**	P < 0.001		P = 0.002	P = 0.462
Not working	1.00		1.00	1.00
Agriculture	0.305 (0.237-0.394)		0.623 (0.481-0.807)	0.864 (0.602-1.241)
Working (paid)	1.077 (0.791-1.466)		0.782 (0.578-1.052)	0.749 (0.471-1.193)
**Paternal education**	P < 0.001		p = 0.042	P = 0.862
No education	1.00		1.00	1.00
Primary	1.497 (1.105-2.028)		1.266 (0.967-1.657)	1.064 (0.754-1.504)
Secondary	3.194 (2.394-4.262)		1.489 (1.129-1.965)	1.146 (0.804-1.635)
Higher	9.244 (6.155-13.887)		1.736 (1.099-2.742)	1.257 (0.665-2.378)
**Paternal occupation**	P < 0.001		P < 0.001	P = 0.768
Agriculture	1.00		1.00	1.00
Professional/Technical	3.990 (3.147-5.059)		1.718 (1.354-2.179)	1.099 (0.851-1.421)
Manual and others	1.704 (1.350-2.151)		1.398 (1.102-1.772)	1.095 (0.578-2.074)
**Proximate factors**				
**Mother’s age (in years)**	P < 0.001			P = 0.294
15-19	1.00			1.00
20-29	0.800 (0.603-1.061)			1.321 (0.858-2.035)
30-34	0.624 (0.452-0.860)			1.420 (0.835-2.416)
> = 35	0.348 (0.246-0.494)			1.580 (0.989-2.524)
**Number of ANC visits**	P < 0.001			P < 0.001
No ANC visit	1.00			1.00
1-3	2.983 (1.983-4.487)			1.597 (1.083-2.355)
4 or more	13.323 (8.570-20.711)			3.624 (2.343-5.604)
**Place of delivery**	P < 0.001			P = <0.001
Home	1.00			1.00
Health facility	49.739 (36.179-68.348)			31.084 (22.416-43.106)
**Maternal final say on her own health care**	P = 0.001			P = 0.587
Women alone	1.00			1.00
Women with partner/other	1.131 (0.910-1.405)			1.143 (0.855-1.530)
Partner alone/someone else	0.765 (0.605-0.966)			1.084 (0.801-1.468)

Model 3: Cox and Snell Psedu R squares: 0.479. Valid cases N = 4079.

Model 1: Place of residence and ecological region. Model 2: place of residence, region, ethnicity, religion, maternal education, maternal occupation, paternal education and paternal occupation. Model 3: place of residence, region, ethnicity, religion, maternal education, maternal occupation, paternal education and paternal occupation, maternal age, number of antenatal visits, place of delivery, maternal autonomy for her own health. P = p- value. 1.00 = reference group. OR = Odd ratio. 95% CI = 95% Confidence interval. ANC: Antenatal care. All values are weighted for the sampling weight, cluster weight and multi stage sampling.

In the final model, when the proximate variables were added to the significant variables of Model 2, place of delivery and antenatal care were significantly associated with postnatal care after controlling for community and sociodemographic factors. Place of delivery had the strongest effect on the attendance of postnatal care showing that mothers who delivered in a health facility (aOR 31.084; 95% CI (22.416-43.106)) were more likely to attend postnatal care than mothers who delivered at home. In addition, mothers who had attended the recommended four or more ANC visits were more likely to attend any postnatal care than the mothers who did not attend any ANC visits (aOR 3.624; 95% CI (2.343-5.604)). Of the community and sociodemographic factors, wealth status and maternal education were the only factors to remain significant in the final model. No association was found between maternal decisions making related to her health with use of any postnatal care.

In an alternative multivariate regression model of postnatal care attendance, the variables place of delivery, number of ANC visits and mother’s age were replaced by the closely related variables of skilled birth attendance, timing of ANC and birth order, respectively (not shown in table). The alternative model yielded similar results to the final model, whereby early attendance to ANC increased the likelihood of postnatal care attendance (aOR1.275; 95% CI (1.005-1.620)). Similarly skilled birth attendance at birth increased the likelihood of postnatal care attendance (aOR33.742 95%CI (26.212-43.434)). No changes in the direction and significance of any other associations differed in the alternative model compared to the final model presented in Table 2.

### Factor associated with immediate postnatal care attendance

The factors associated with immediate postnatal care are presented in Table 3.In Model 1, community factors: place of residence and ecological regions were significant showing that mothers from urban (aOR 3.539; 95% CI (2.725-4.736)) and mothers from Terai (Plain) region (aOR 2.474; 95% CI (1.680-3.647)) were more likely to attend immediate postnatal care than mothers living in rural and mountain areas, respectively. When sociodemographic factors were added (Model 2), both of these factors remained significant. In Model 2, wealth status, maternal education, maternal occupation, paternal education and paternal occupation were significantly associated with immediate postnatal care service utilisation after controlling for community factors, religion and ethnicity. Similar to the utilisation of any postnatal care; mothers from rich family, with higher education, having their partner higher education were more likely to use immediate postnatal care. Likewise, mothers with an agriculture occupation were less likely to attend immediate postnatal care. Mothers who had partners from professional and manual occupations were more likely to attend immediate postnatal care.

**Table 3:** Factors associated with attending immediate postnatal care among Nepalese women, Demographic and Health Survey 2011

**Factor**	**Unadjusted OR (95% CI)**	**Adjusted OR (95% CI)**		
		**Model 1**	**Model 2**	**Model 3**
**Community factors**				
**Place of residence**	P < 0.001	P < 0.001	P = 0.006	P = 0.227
Rural	1.00	1.00	1.00	1.00
Urban	3.511 (2.714-4.511)	3.539 (2.725-4.739)	1.464 (1.116-1.921)	0.777 (0.515-1.171)
**Ecological region**	P < 0.001	P < 0.001	P = 0.026	P = 0.086
Mountain	1.00	1.00	1.00	1.00
Hill	1.682 (1.114-2.539)	1.461 (0.960-2.223)	1.047 (0.723-1.517)	0.939 (0.668-1.321)
Terai	2.684 (1.828-3.941)	2.474 (1.680-3.647)	1.473 (1.001-2.171)	1.313 (0.921-1.873)
**Sociodemographic factors**				
**Wealth status**	P < 0.001		P < 0.001	P = 0.001
Poor (Lower 40%)	1.00		1.00	1.00
Middle (Middle 40%)	2.543 (2.009-3.219)		1.663 (1.272-2.174)	1.670 (1.111-2.512)
Rich (Upper 20%)	6.609 (5.143-8.843)		3.068 (2.106-4.470)	3.693 (2.319-5.880)
**Ethnicity**	P < 0.001		P = 0.0519	Not in model
Advantaged	1.00		1.00	
Disadvantaged (Janjati)	0.566 (0.443-0.723)		0.888 (0.677-1.166)	
Disadvantaged (Dalit)	0.526 (0.420-0.657)		1.076 (0.832-1.392)	
**Religion**	P = 0.014		P = 0.350	Not in model
Hindu	1.477 (1.081-2.017)		1.167 (0.844-1.613)	
Others	1.00		1.00	
**Maternal education**	P < 0.001		P < 0.001	P = 0.502
No education	1.00		1.00	1.00
Primary	1.852 (1.477-2.323)		1.483 (1.193-1.843)	1.141 (0.825-1.576)
Secondary	4.333 (3.436-5.464)		2.287 (1.789-2.923)	1.189 (0.849-1.665)
Higher	12.824 (8.404-19.567)		3.657(2.273-5.882)	1.563 (0.819-2.982)
**Maternal occupation**	P < 0.001		P < 0.002	P = 0.396
Not working	1.00		1.00	1.000
Agriculture	0.318 (0.252-0.402)		0.644 (0.506-0.819)	0.936 (0.661-1.326)
Working (paid)	1.049 (0.784-1.403)		0.776 (0.586-1.027)	0.730 (0.458-1.164)
**Paternal education**	P < 0.001		P = 0.036	P = 0.781
No education	1.00		1.00	1.00
Primary	1.509 (1.101-2.069)		1.272 (0.953-1.699)	1.044 (0.717-1.520)
Secondary	3.229 (2.390-4.363)		1.521 (1.134-2.038)	1.150 (0.812-1.628)
Higher	8.854 (5.886-13.321)		1.817 (1.123-2.939)	1.293 (0.963-2.403)
**Paternal occupation**	P < 0.001		P < 0.001	P = 0.890
Agriculture	1.00		1.00	1.00
Professional/technical	3.856 (3.039-4.892)		1.677 (1.302-2.160)	1.070 (0.812-1.411)
Manual and others	1.699 (1.343-2.150)		1.396 (1.101-1.769)	1.062 (0.553-2.040)
Proximate factors				
**Mother’s age (in years)**	P < 0.001			P = 0.350
15-19	1.00			1.00
20-29	0.816 (0.619-1.077)			1.426 (0.941-2.161)
30-34	0.646 (0.472-0.884)			1.568 (0.925-2.658)
> = 35	0.335 (0.239-0.471)			1.399 (0.883-2.217)
**Number of ANC visits**	P < 0.001			P < 0.001
No ANC visit	1.00			1.00
1-3	3.019 (1.934-4.712)			1.670 (1.111-2.512)
4 or more	13.325 (8.274-21.457)			3.693 (2.319-5.880)
**Place of delivery**	P < 0.001			P < 0.001
Home	1.00			1.00
Health facility	52.920 (38.559-72.630)			34.894 (24.956-48.789)
**Maternal final say on her own health care**	P < 0.001			P = 0.665
Women alone	1.00			1.00
Women with partner/other	1.114 (0.898-1.381)			1.082 (0.783-1.495)
Partner alone/someone else	0.729 (0.575-0.923)			0.940 (0.961-1.278)

Valid cases N = 4079; Cox and Snell Pseudo R Squares = 0.480; Model 1: Place of residence and ecological region. Model 2: place of residence, region, ethnicity, religion, maternal education, maternal occupation, paternal education and paternal occupation. Model 3: place of residence, region, ethnicity, religion, maternal education, maternal occupation, paternal education and paternal occupation, maternal age, number of antenatal visits, place of delivery, maternal autonomy for her own health. P = p- value. 1.00 = reference group. OR = Odd ratio. 95% CI = 95% Confidence interval. ANC: Antenatal care. All values are weighted for the sampling weight, cluster weight and multi stage sampling.

In the final model (Model 3), when proximate variables were added to Model 2, wealth status, number of ANC visits and place of delivery were significantly associated with immediate postnatal care. Similar to any postnatal care, the place of delivery had strongest effect on immediate postnatal care attendance.

In an alternative multivariate regression model of immediate postnatal care, the variables place of delivery, number of ANC visits and mother’s age were replaced by the closely related variables of skilled birth attendance, timing of ANC and birth order, respectively (Not shown in table). This alternative model yielded comparable results to the final model of immediate postnatal care, whereby early attendance to ANC was marginally associated with immediate postnatal care (p = 0.057) showing that the mothers who attended ANC early were more likely to have immediate postnatal care (aOR 1.272; 95% CI (0.993-1.630)). While skilled birth attendance at birth increased the likelihood of immediate postnatal care attendance (aOR 45.109; 95%CI (34.102-59.668). No changes in the direction and significance of any other associations differed in the alternative model compared to the final model presented in Table 3.

## Discussion

This study has found that utilisation of postnatal care has increased between 2006 to 2011 from 26.5% [13] to 43.2%. Nevertheless, utilisation of postnatal care and immediate postnatal care among Nepalese mothers remains low. Community factors (place of residence and ecological region), socio-demographic factors (wealth status, religion, education, maternal occupation) and proximate factors (the use of recommended ANC service, place of delivery, skilled attendance during delivery) were significantly associated with the utilisation of postnatal care. A possible explanation for the low attendance to postnatal care in Nepal, could be cultural practice which prevents recently delivered mothers and newborns to be touched by any one or leave the house until the 12^th^ day after delivery [29]. Such cultural practices have been reported in Bangladesh and India previously [30,31] and have been associated with non-utilisation of postnatal care [31].

At the community level, the quality of care may also be preventing women from attending postnatal care. In Nepal, despite a focus on community-based services through primary health care outreach clinics, these clinics have been reported to function at a limited capacity [32,33], where lowly trained village health workers who have only a few months of training, and do not have a high school level qualification are often found running such clinics [32]. The major focus of their job is child immunisation. As result, they are rarely provided with postnatal care training. While these clinics can generally reach vast number of women, the clinics are usually conducted in public places such as schools, waiting areas of community centres, and temples; and thus lack adequate space for privacy. This lack of privacy, technical competency of the health workers, and lack of equipment may be the reason for the low utilisation of postnatal care by mothers.

One of the encouraging findings of this study is the effect of ANC attendance on postnatal care attendance. This study found that the mothers who attended four or more ANC visits as recommended by the WHO [1] and the National guidelines of Nepal [32], were more likely to attend postnatal care. ANC attendance and adequate counselling of mothers has been previously reported to be associated with postnatal care attendance [34].

Place of delivery was significantly associated with attending immediate postnatal care. Women who deliver in a health facility, receive medical care from skilled attendants. Subsequently, mothers should receive postnatal care from skilled attendants within 24 hours after birth, before being discharged from the place of delivery, as per the Nepal postnatal guidelines [35]. Interestingly, this was not fully true for one in ten mothers in this study. Missing the opportunities to provide essential postnatal care in facility deliveries unnecessarily puts the lives of mothers and infants at risk. Future research needs to explore why such opportunities to provide postnatal care to mothers who deliver in health facilities and deliveries attended by skilled attendant are missed.

Mothers from urban areas and mothers from Terai areas were more likely to attend postnatal within 42 days after delivery and immediate postnatal care. This finding can be explained by the fact that in the mountainous and rural areas of Nepal there has less access to public services, such as roads, transport and health services. As a result, urban and Terai residents are likely to have more access to transportation and healthcare services [36]. A higher physical accessibility has been previously found to increase maternal health service utilisation in Nepal [15] and Ghana [37]. This finding suggests there is a need to provide postnatal care through alternative means.

The finding that mothers with higher education were more likely to attend postnatal care can be explained by the notion that mothers with higher levels of education are more likely to be informed about health risks, demand and gain access to healthcare [34]. Likewise, mothers who are involved in paid employment are more likely to be economically independent and consequently have access to services, and utilise the services when they need or as recommended by their health workers [10]. Previous studies by Simkhada et al. [38], Neupane et al. [15] and Salam et al. [39] also support the notion that attainment of education, and having a paid job empowers mothers to utilise maternal health services.

With regard to employment sector, agriculture is a very significant contributor to Nepal’s economy, with almost three in four households involved in agricultural activities, primarily as subsistence agriculture [5]. It is reasonable to assume that households who entirely depend on agriculture may be unable to attend postnatal care due to time away from work affecting their food production and income. Opening hours of health facilities (usually operating between 10.00 am-3.00 pm or only on certain days) may discourage women from seeking care. This conflicting time schedule may provide an explanation for the decreased likelihood of postnatal care attendance among mothers reporting agricultural occupation.

The current study also supported the common finding that mothers from rich households are more likely to attend postnatal care and immediate postnatal care. This finding can be explained by the availability of funds to spend on hospital for deliveries and obtain subsequent services such as postnatal care. Likewise, mothers from higher socioeconomic households are also more likely to be aware of the benefits of obtaining postnatal care through different media such as television, and newspapers than their counterparts from low socioeconomic groups. This finding is similar to the findings from the India and Nepal where mothers from higher socioeconomic group attend postnatal services [12,13,34,40,41].

### Public health implication

This study highlights the need to increase the availability and accessibility of health facilities during pregnancy, delivery and postnatal periods, as utilisation of ANC and delivery services increased the likelihood of postnatal care utilisation. Results from this study point to a combination of community and facility-based interventions as feasible measures to reach poor, rural, and less educated mothers including all mothers of the community [9]. To increase service use, it is necessary to invest in the existing networks of community health workers who run out-reach clinics in order to strengthen their capacity to provide quality maternity care with the necessary knowledge, skills and equipment. Such initiatives will ensure the most disadvantaged groups and those who cannot travel to health facilities due to long hours of work in the agricultural sector and those who live in remote hard-to-reach areas receive the necessary maternity care.

This study found that a significant proportion of the mothers who delivered in health facility or were attended by a skilled personal during delivery did not receive postnatal care. An incentive program to cover the transportation cost of mothers and incentive for health workers has been found successful in increasing the use of institutional deliveries [9,32]. A supply-side scheme, which rewards health workers for each postnatal woman they see, could be an option to ensure that all women who deliver in a health facility receive postnatal care.

Approximately 85% of mothers in Nepal attend at least one ANC visits, while the proportion of mothers attending four or more ANC visits drops to 50% [32]. This is a critical figure and, it suggests that health professionals should take advantage of the first ANC visit to highlight the potential risks of giving birth at home and not attending postnatal care. Further, as results suggest, encouraging mothers to attend the recommended four or more ANC visits will have a positive effect of postnatal care uptake. Providing education to husbands during antenatal period [42], and increasing awareness about attending the recommended four ANC visits [19] can have a positive effect in increasing institutional deliveries and postnatal care. At the community level, involvement of community leaders including religious leaders in health programs may be helpful in increasing the utilisation of postnatal care during the seclusion period when the mother and infant are confined to the isolation within their own house [30]. While it is encouraging to see the current implementation of community-based newborn programs focusing on the provision of home-based care to provide optimum care for newborn [43], future pilot studies may evaluate the effect and feasibility of a similar approach to increase the use of postnatal care.

### Strengths and limitations

The current study has a number of strengths. We used a national survey data, and relatively large sample size with a high response rate (95%) [5]. The demographic and health surveys are internationally validated and nationally adapted surveys. Therefore, the current findings are generalisable to the entire country. Our analysis accounted for study design and sampling procedure, which is more likely to yield accurate estimates [26]. This study has also provided updated knowledge on factors associated with the utilisation of postnatal care. In addition, this is the first study to report the prevalence and determinants of immediate postnatal care in Nepal. Nevertheless, the current study has several limitations. Cross-sectional nature of NDHS limits the capacity to draw any causal inferences. Also, as the survey asked the information retrospectively, this may have yielded some recall bias. Nevertheless, this bias is not considered problematic since this study included only women giving birth within five years preceding the survey.

## Conclusion

The current study found that less than a half of mothers utilised postnatal care in Nepal. Promoting ANC, health facility delivery and postnatal care, focusing on women residing in rural areas, and less educated mothers is essential to increase the use of postnatal care and thereby decrease maternal and new-born morbidity and mortality. Furthermore, strengthening the current capacity of facilities and outreach clinics to enable the provision of quality service may help to increase the utilisation of postnatal service [9]. Upgrading the skills of the community health workers and raising awareness about the availability and importance of postnatal care through outreach clinics could also be a feasible option to bring about an increase in postnatal care uptake, even in a short time period. Further operational research is essential to explore feasible options for community-based services to increase the use of postnatal care in Nepal. Current community-based maternal and child health program could be a good point to start to integrate postnatal care as a mandatory part of care of mother and newborn in community [44,45].

## Competing interest

We declare that we have no competing interest.

## Authors’ contribution

MA and VK jointly contributed to the concept of the study. VK performed statistical analysis; VK and MA jointly wrote the draft of manuscript. RK and TG contributed in interpreting the result, literature review and revising the manuscript. All authors revised the manuscript and agreed on the findings and views expressed.

## Authors’ information

VK is independent consultant. He holds Master of public health. He worked in maternal and child health programs in Nepal. MA is a program officer in Women’s Health Project implemented by Population Services International in Nepal. She hold Bachelor’s degree in public health. RK is an assistant professor in School of Public Health and Community Medicine, BP Koirala Institute of Health Sciences. His area of research is focussed around maternal health. TG is PhD student and holds a Master’s Degree in International Health. Her research area includes maternal and child health focusing on urban poor communities.

## Pre-publication history

The pre-publication history for this paper can be accessed here:

http://www.biomedcentral.com/1472-6874/14/19/prepub
